# Comparison of Combined General-Epidural Anesthesia with General Anesthesia Effects on Survival and Cancer Recurrence: A Meta-Analysis of Retrospective and Prospective Studies

**DOI:** 10.1371/journal.pone.0114667

**Published:** 2014-12-30

**Authors:** Lijian Pei, Gang Tan, Lei Wang, Wenjuan Guo, Bo Xiao, Xianli Gao, Li Wang, Hong Li, Zhonghuang Xu, Xiuhua Zhang, Jing Zhao, Jie Yi, Yuguang Huang

**Affiliations:** 1 Department of Anesthesiology, Peking Union Medical College Hospital, Chinese Academy of Medical Sciences, Beijing 100730, PR China; 2 Department of Epidemiology & Biostatistics, Institute of Basic Medical Sciences, Chinese Academy of Medical Sciences & School of Basic Medicine Peking Union Medical College, Beijing 100005, PR China; Imperial College London, Chelsea & Westminster Hospital, United Kingdom

## Abstract

**Objective:**

Animals underwent combined general-epidural anesthesia (EGA) is reported to have better long-time outcome than general anesthesia (GA). This study aimed to make overall evaluation of the association between these two anesthetic techniques and prognosis of cancer patients undergoing surgery.

**Methods:**

Related databases such as PubMed and EMbase were searched for eligible studies that evaluated the influence of EGA and GA on the prognosis of cancer patients undergoing surgery. Selected studies were evaluated according to the inclusion criteria by two reviewers respectively, followed by data extraction and quality assessment. The odds ratio (OR) with their 95% confidence intervals (CIs) were calculated to assess the influence strength of EGA and GA on prognosis of cancer patients.

**Results:**

A total of ten studies involving 3254 patients were included. The overall results demonstrated that there was no significant difference between EGA and GA group (OR = 0.88, 95% CI 0.73 to 1.06, P = 0.187) concerning postoperative recurrence and metastasis rate. In regard to the following two factors: cancer category and time of follow-up, subgroup analysis identified significant differences between EGA and GA in the group of patients with prostate cancer and the group with follow-up less than or equal to two years (OR = 0.66, 95% CI 0.46 to 0.95, P = 0.027; OR = 0.70, 95% CI 0.51 to 0.98, P = 0.035; respectively) concerning postoperative recurrence and metastasis rate. However, no significant difference was found in the group of patients with colorectal cancer (OR = 1.06, 95% CI 0.84–1.33, P = 0.62).

**Conclusions:**

This meta-analysis showed that EGA might be associated with improvement in prognosis of patients with operable prostate cancer and the cancer patients with follow-up less than or equal to two years. However, no obvious relationship between the improvement in prognosis of colorectal cancer and EGA were detected, comparing to GA. Furthermore, all the results should be interpreted cautiously, as heterogeneous data were used for analyzing.

## Introduction

Recently, considerable numbers of studies have reported that the anesthetic technique applied during oncologic surgery could affect the recurrence and metastasis of cancer, for example, combined general and regional anesthesia is proved to be able to improve the postoperative prognosis of prostate cancer (epidural analgesia) and breast cancer (paravertebral block) [1], [2]. The potential bases of this intriguing hypothesis lies on the effect of anesthetic technique or specific anesthetic drug on the immune response and tumor cell biology in cancer patients, which may consequently affect the oncological outcome, including recurrence and metastasis after surgery [3].

Epidural anesthesia (EA) could reduce the incidence of side-effects and the occurrence possibility of immunosuppressive factors, as well as provide better postoperative pain relief [4]. Moreover, animal models also demonstrated that EA would significantly attenuate the stress response during surgery through preserving immune response ability, leading to better long-time outcome [5]. There is a hypothesis that perioperative EA could improve perioperative immune suppression and enhance immune surveillance in patients with cancer, and thus reduce cancer recurrence and metastasis. Previous retrospective studies on breast and prostate cancer patients have supported this hypothesis [6], [7]. However, some recent retrospective studies showed that EA could not bring obvious improved oncologic outcomes in patients with prostate, colorectal, and breast cancers [8]–[13].

The combined general-epidural anesthesia (EGA) is frequently applied in major thoracic or abdominal surgery [14]. Nizamoglu et al demonstrated that EGA performed on adrenal tumor patients undergoing laparoscopic adrenalectomy was a relatively effective and safe anesthetic method, which could help maintain normal hormone level [15]. Kaneda et al showed that the blood pressure of patient with pheochromocytoma could be effectively controlled with the use of EGA [16]. Sotunmbi et al found that patients needed less medication and their hemodynamics was stable after surgical resection of giant pheochromocytoma by using EGA [17]. As for larynx or hypopharynx and gastro-esophageal cancer, EGA was proved to contribute in the significantly reduced cancer recurrence and prolonged cancer-free survival [18], [19]. Nevertheless, Wuethrich et al showed that there was no significant survival improvement in prostate cancer patients who underwent retropubic radical prostatectomy using EGA [15]. Due to the limited sample size and other variation factors, the results of these publications are conflicting rather than conclusive. In order to provide further evidence that the combination of EA and GA might be related with decreased likelihood of cancer recurrence after tumor resection and thus improve overall survival, we conducted this comprehensive meta-analysis.

## Materials and Methods

### Literature search

Related databases such as PubMed, EMbase were searched for relevant studies using the following key words: “anesthetic technique” or “general anesthesia” or “epidural anesthesia” or “regional anesthesia”, and “cancer” or “carcinoma” or “neoplasm”, and “survival” or “recurrence” or “metastasis”. The last computerized literature search was in March 2014 and without language restriction. Bibliographies of retrieved publications were also examined for additional literatures. We first identified 585 potentially relevant papers with this search strategy, and then screened 290 possibly eligible papers by reviewing their titles. After scanning the abstract, 26 studies were identified for further screening by reading the full text.

### Criteria for inclusion

The publications included in our meta-analysis should meet the following criteria: 1) they were independent prospective or retrospective case-control studies; 2) the effect of combination of EA and GA on oncological outcome (cancer-free survival or cancer recurrence or metastasis) was used to compare with that of GA alone in cancer surgery; 3) studies showed sufficient useful data to calculate odds ratio (OR) with its 95% confidence intervals (CI). Reviews, meta-analyses and the studies did not provide sufficient data were excluded. After reading full text of all the possibly eligible publications, we finally identified 10 studies for our comprehensive meta-analysis.

### Quality assessment

Two experienced investigators (LJP and GT) independently evaluated the quality of the eligible studies based on the detailed data of included studies. Any discrepancy was subsequently settled by consultation. The quality of studies was assessed and quantified using the 9-star Newcastle-Ottawa Scale [20]. Besides, we followed the published PRISMA statement for reporting systematic reviews and meta-analyses of studies that evaluate health care interventions [21].

### Data extraction

Two experienced investigators (LJP and GT) independently performed the data extraction. The following detailed data were collected from each study: the surname of first author, the year of publication, cancer type, design type, survival, numbers in EGA, numbers in GA and numbers of cancer patients with recurrence or metastasis.

### Statistical analysis

In this meta-analysis, the software Stata 12.0 (Stata Corporation, College Station, TX, USA) was used to pool the results of eligible studies. The OR with its 95% CI was used to evaluate the influence strength of EGA and GA on the prognosis of cancer patients for each study. The heterogeneity among the independent studies was evaluated by Q statistics and I^2^ statistics, and it is considered to be heterogeneous if P-value<0.05 or I^2^>50% [22]. The fixed effects model would be adopted if there was no heterogeneity among studies, otherwise, the random effects model would be used. We used Begg’s funnel plot and Egger’s linear test to visually examine the potential publication bias. A symmetrical plot and P-value of Egger’s test (>0.05) suggested that there was no publication bias in all studies. To make a comprehensive analysis, we also conducted stratified analysis by cancer type and the follow-up years. Sensitivity analysis was performed by omitting one study each time to detect extreme values.

## Results

### Basic characteristics

**Figure 1:**
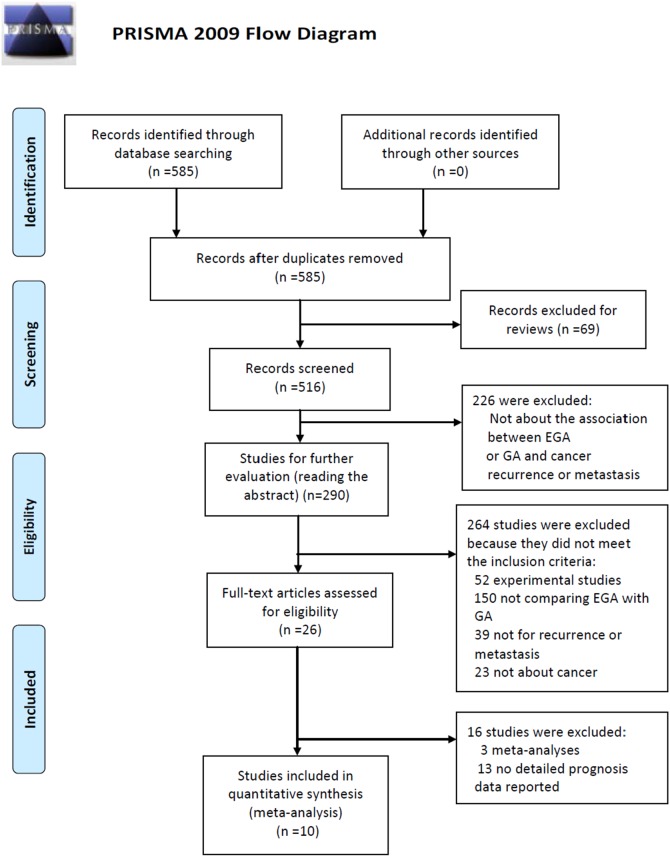
Flowchart of included and excluded studies.

After comprehensive search in the databases, we finally identified 10 eligible studies, including 1351 cases in the EGA group and 1903 cases in the GA group [1], [2], [6], [11], [13], [15], [23]–[26]. The flowchart for literature search is displayed in Fig. 1. Cancer recurrence or metastasis was considered as the end point event. The basic characteristics of studies are shown in Table 1 and Table 2. The quality of each study was graded as level 2 (6 to 9) according to the 9-star Newcastle-Ottawa Scale. Four types of cancer such as breast cancer, prostate cancer, colorectal cancer and gastro-esophageal cancer were included in this study. Four studies were with regard to colorectal cancer, four studies were concerned with prostate cancer, with the other two literatures was related to breast cancer and gastro-esophageal cancer, respectively.

**Table 1:** Basic characteristics of included studies.

Authors	Cancer type	Design type	Survival	Quality*
Exadaktylos (2006)	Breast cancer	Retrospective	Recurrence-freesurvival	7
Biki (2008)	Prostate cancer	Retrospective	Recurrence-freesurvival	6
Christopherson (2008)	colon cancer	Prospective	Overall survival	7
Gottschalk (2010)	Colorectal cancer	Retrospective	Recurrence-free survival	7
Luo (2010)	colon cancer	Retrospective	Recurrence-free survival	7
Tsui (2010)	Prostate cancer	Prospective	Disease-free survival	6
Wuethrich (2010)	Prostate cancer	Retrospective	Progression-freesurvival	7
Gupta (2011)	Colon cancer	Retrospective	Overall survival	6
Wuethrich (2013)	Prostate cancer	Retrospective	Overall survival	7
Hiller (2014)	Gastro-esophagealcancer	Retrospective	Overall survival	7

*evaluated by the 9-star Newcastle-Ottawa Scale; EGA: epidural anesthesia combined with general anesthesia; GA: general anesthesia.

**Table 2:** Basic characteristics of included studies (Number of events).

Authors	Events (n)	EGA (n)	Events (n)	GA (n)
Exadaktylos (2006)	3	50	19	79
Biki (2008)	15	102	43	123
Christopherson (2008)	45	85	56	92
Gottschalk (2010)	34	256	40	253
Luo (2010)	42	182	155	931
Tsui (2010)	11	49	17	50
Wuethrich (2010)	12	103	20	158
Gupta (2011)	129	562	20	93
Wuethrich (2013)	14	67	18	81
Hiller (2014)	32	97	17	43
Follow-up less than 2 years				
Exadaktylos (2006)	3	50	19	79
Biki (2008)	1	102	5	123
Christopherson (2008)	11	73	15	79
Gottschalk (2010)	34	256	40	253
Tsui (2010)	3	49	5	50
Hiller (2014)	32	97	17	43

Events: cancer recurrence or metastasis.

### Quantitative data synthesis

### • Association between EGA and overall cancer recurrence or metastasis

**Figure 2:**
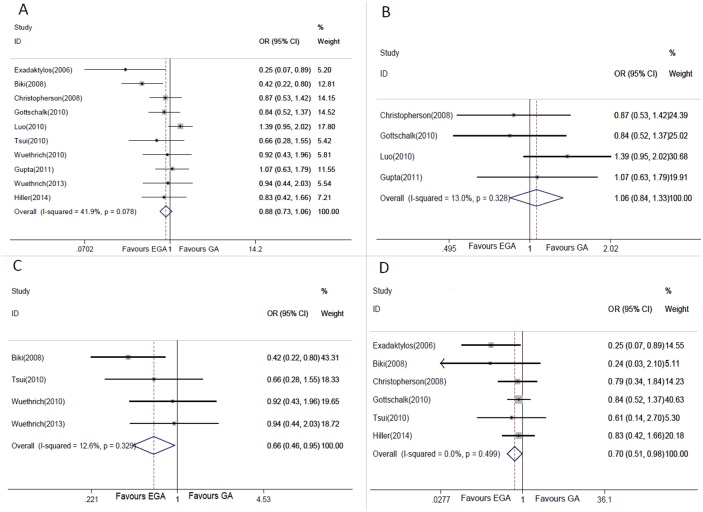
Forest plots showing the association between EGA and cancer recurrence or metastasis. A. overall cancer; B. colorectal cancer; C. prostate cancer; D. cancer with follow-up less than or equal to two years.

Overall recurrence rates for cancer patients were 21.70% (337/1553) and 21.28% (405/1903) in EGA and GA group respectively. No apparent heterogeneity was detected (chi-squared = 15.50, P = 0.078, I^2^ = 41.9%), so fixed effects model was used to calculate the combined OR and 95% CI. The pooled results demonstrated that EGA did not decrease cancer recurrence or metastasis in comparison with GA alone (OR = 0.88, 95% CI = 0.73–1.06, Z = 1.32, P = 0.187, Fig. 2A).

### • Association between EGA and colorectal cancer recurrence or metastasis

As there were different types of cancer were included in this study, stratified analysis was performed. Overall recurrence or metastasis rates for colorectal patients were 23.04% (250/1085) and 19.80% (271/1369) in EGA and GA group respectively. No significant heterogeneity was found (chi-squared = 3.45, P = 0.328, I^2^ = 13.0%), and fixed effects model was utilized to calculate the pooled OR and 95% CI. The combined results suggested that EGA did not reduce the colorectal cancer recurrence or metastasis in comparison with GA alone (OR = 1.06, 95% CI = 0.84–1.33, Z = 0.50, P = 0.620, Fig. 2B).

### • Association between EGA and prostate cancer recurrence or metastasis

Total 4 eligible studies reported the prognosis data of prostate cancer patients, which was used for the stratified analysis. Overall recurrence or metastasis rates for patients with prostate cancer were 16.20% (52/321) and 23.79% (98/412) for EGA and GA group respectively. No significant heterogeneity was found (chi-squared = 3.43, P = 0.329, I^2^ = 12.6%), and fixed effects model was used to conduct this meta-analysis. The overall results suggested that EGA could obviously decrease the prostate cancer recurrence or metastasis compared with GA alone (OR = 0.66, 95% CI = 0.46–0.95, Z = 2.21, P = 0.027, Fig. 2C).

### • Association between EGA and recurrence or metastasis of cancer with follow-up less than or equal to two years

As is known, complications usually turn up within 2 years post-surgery. Therefore, to investigate the short term effect of anesthesia on survival, it is necessary to conduct a subgroup analysis of patients with less than or equal to two years follow-up. The overall recurrence or metastasis rates for cancer patients with follow-up less than or equal to two years were 13.40% (84/627) and 16.11% (101/627) for EGA and GA group respectively. No heterogeneity was found among all studies (chi-squared = 4.36, P = 0.499, I^2^ = 0%), and fixed effects model was adopted. The pooled results demonstrated that EGA could significantly decrease the cancer recurrence or metastasis with follow-up less than or equal to two years in comparison with GA alone (OR = 0.70, 95% CI = 0.51–0.98, Z = 2.11, P = 0.035, Fig. 2D).

### Sensitivity analysis

**Figure 3:**
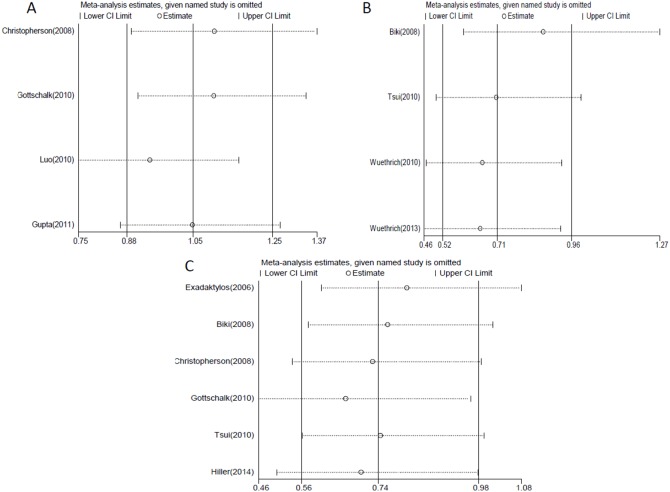
Sensitivity analyses for included studies. A. all studies concerning colorectal cancer; B. all studies concerning prostate cancer; C. studies with follow-up less than or equal to two years.

The root of heterogeneity was assessed by sensitivity analysis, and no apparent heterogeneity was detected as described above. Besides, the pooled OR was not affected by any individual study, as shown in sensitivity analyses, which demonstrated that the results of our meta-analysis were robust and stable (Fig. 3).

### Publication bias

**Figure 4:**
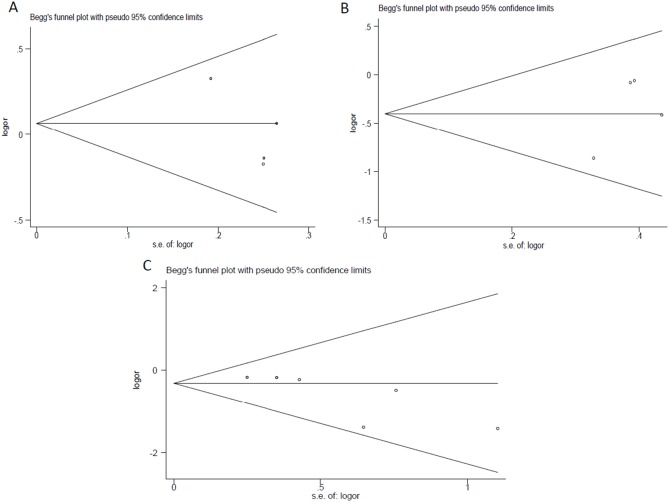
Begg’s funnel plots for publication bias test for studies in subgroups. A. colorectal cancer group; B. prostate cancer group; C. group with follow-up less than or equal to two years.

We examined publication bias through graphical inspection and quantitative evaluation. Neither Begg’ funnel plot nor Egger’s test demonstrated the presence of publication bias in colorectal cancer group (Begg’s test P = 1.000; Egger’s test t = –2.21, P = 0.158) (Fig. 4A), prostate cancer group (Begg’s test P = 0.734; Egger’s test t = 1.32, P = 0.318) (Fig. 4B), and group with follow-up less than or equal to two years (Begg’s test P = 0.060; Egger’s test t = –2.61, P = 0.059) (Fig. 4C).

## Discussion

This meta-analysis was not able to prove a favorable correlation between EGA andoverall cancer recurrence. The same result was found in subgroup analysis of colorectal cancer. However, within the subgroup of prostate cancer patients and subgroup with follow-up less than or equal to two years, an effective perioperative anesthesia combing epidural with general was revealed to be associated with cancer-free survival benefit. Our results from comprehensive meta-analysis were in coincidence with most, even not all, results from recently reported studies [18], [27]. These results are crucial considerations for subsequent prospective studies investigating the intriguing prospect of appropriate perioperative EGA modifying cancer recurrence.

Although cancer recurrence and metastasis may be associated with immunosuppressive factors such as anesthetic drugs, opioids and surgical stress, EA may promote tumor surveillance with less systemic immunosuppressive factors exposure [12]. It is well known to all, immune suppression may assist the expansion of minimal residual disease after surgical resection, with the persistence of dormant tumor cells or circulating tumor cells, which will finally result in cancer recurrence and counteracted beneficial effect of surgery. However, Myles et al reported that EA in abdominal malignancies surgery was not associated with enhanced recurrence-free survival [28], and the similar result has been found in another two studies [27], [29]. Intriguingly, systematic results from population-based cohort studies and randomized clinical trials of other noncancerous surgeries suggested that EA might decrease the mortality, morbidity, and other severe complications when it is compared with GA [30]–[33]. The beneficial effect of EA on decreased cancer-recurrence and metastasis may be in accordance with the hypothesis that there is disturbance of immune defense mechanisms in patients undergoing cancer surgery, for example, neutrophils from patients undergoing surgery showed improved movement under spinal anesthesia when compared with GA [34]. Other possible explanation covers hemodynamic instability or compromise, surgical stress response, pain provocation, and recovery of spontaneous respiratory. During the inflammation, numerous mediators produced by leucocytes and endothelial cells will elicit pain, which can be counteracted by the opioid peptides in the peripheral nerve terminals [35]. Inflammatory reactions rise in the destructed tissues, leading to the activation of pain receptors [36]. Immune compromise may exert effect on the rate of postoperative infection, duration of wound healing, treatment response, and tumor cell dissemination. Besides, surgical procedures and anesthesia techniques have been proved to be able to inhibit the activity of natural killer (NK) and functional T cells for several days [37]–[39]. Furthermore, in tumor microenvironment, Th1/Th2 balance is destroyed. Zhou et al demonstrated that EA is remarkably superior to GA in reversing this immunodeviation, and potentially exerts beneficial effect on hepatocellular cancer patients by enhancing anti-tumor Th1 polarization [40]. Due to the use of volatile anesthetics and opioids, the humoral and cellular immunity are suppressed, and thus the angiogenesis and micro-metastases occur. But propofol anesthesia reshapes the antitumoral effects by reducing the production of cyclooxygenase 2 and prostaglandins E2 from cancer cells [41]. Therefore, in order to improve clinical outcome of cancer patients, it is a practical strategy to preserve the immune function by using individualized therapeutic methods. The EGA method may contribute to the intact and sustained immunological function by reducing the dosage of anesthetic agents and thus lead to a better clinical outcome.

Nevertheless, since invasive techniques are being adopted minimally in general, and EA is being utilized less frequently, large scale randomized controlled clinical trials are unlikely to be performed to verify our theory. Generally speaking, the indications for EA have shrunk, and are restricted to certain high-risk patients, thoracotomy, major vascular and major upper abdominal surgery [42]. In our meta-analysis, there was no association between EGA and decreased overall cancer recurrence and metastasis, even in subgroup analysis for colon cancer. However, this association was significant within the subgroup of prostate cancer patients and subgroup with follow-up less than or equal to two years. Moreover, surgical outcome could be affected by multiple factors, so it is difficult to draw a conclusion for all cancer types and our hypothesis should be explained with caution, and there is no obvious evidence that simple alternation in the anesthesia practice would have a remarkable positive effect on postoperative survival of patients with cancer. What’s more, considering the grossly heterogeneous data used across a range of different cancers, all findings should be interpreted cautiously. More prospective studies including standardized cancer patients, larger numbers of cases, unified anesthetic techniques, and systematic long-term follow-up are needed to demonstrate whether the association of EGA with decreased cancer recurrence and metastasis is causative.

## Supporting Information

S1 PRISMA Checklist 10.1371/journal.pone.0114667.s001
(DOC)
